# 

**Published:** 1887-08

**Authors:** 


### CONTENTS, AUGUST, 1887, VOL. 3, NO. 2.

ORIGINAL COMMUNICATIONS:—

Hemorrhage of the Mouth in an Infant 9 days old, Stomatitis Parasitica; R. Menger, M. D.......... 35

An Ugly Enterocele, Operation and Cure; F. H. Tucker, M. D.......... 40

A Country Doctor's First Case of Surgery; R. H. Day, M. D. 42

An Enemy of the Bovines attacks a Boy; G. W. Christian, M. D.......... 45

Another Case of Maggots in the Nasal Cavity; F. A. Schmitt, M. D.......... 46

SOCIETY NOTES:—

(Original Communication) On the Conduct of Medical Societies; D. R. Wallace, M. D.......... 47

More about the International Medical Congress and Railroad Rates.......... 54

Transactions Texas State Medical Association, '87.......... 55

To the Congress.......... 55

EDITORIAL:—

The State Lunatic Assylum and its Management.......... 56

MEDICAL NEWS AND MISCELLANY:—

Many items.......... 60

BIBLIOGRAPHY.......... 66

ADVERTISERS' NOTICES.......... 73

